# μ-Oxido-bis­[(5,10,15,20-tetra­phenyl­porphyrinato-κ^4^ *N*,*N*′,*N*′′,*N*′′′)manganese(III)]

**DOI:** 10.1107/S2414314622008690

**Published:** 2022-09-02

**Authors:** Wanjie Ren, Jianfeng Li

**Affiliations:** a College of Materials Science and Opto-electronic Technology, CAS Center for Excellence in Topological Quantum Computation & Center of Materials Science and Optoelectronics Engineering, University of Chinese Academy of Sciences, Yanqi Lake, Huairou District, Beijing 101408, People’s Republic of China; Vienna University of Technology, Austria

**Keywords:** Manganese porphyrin, μ-oxido bridging mode, crystal structure

## Abstract

In the crystal structure of the title oxido-bridged binuclear complex, [Mn^III^(TPP)]_2_O (TPP = tetra­phenyl­porphyrinate, C_44_H_28_N_4_) or [Mn_2_(C_44_H_28_N_4_)_2_O], the two penta­coordinate manganese(III) ions are bridged by a single oxido ligand, with an Mn—O distance of 1.7600 (3) Å and an Mn—O—Mn bridging angle of 176.1 (2)°. The bridging O^2−^ ligand is located on a twofold rotation axis, resulting in point group 2 for the entire complex. The Mn^III^ atom is displaced out of the 24-atom mean plane of the porphyrine entity by 0.52 Å. C—H⋯π and π–π inter­actions help to stabilize the mol­ecular packing within the crystal structure.

## Abstract 2

The oxido-bridged dinuclear complex, [Mn(TPP)]_2_O, has an Mn—O distance of 1.7600 (3) Å, an Mn—O—Mn bridging angle of 176.1 (2)°, and exhibits point group symmetry 2.

## Graphical abstract

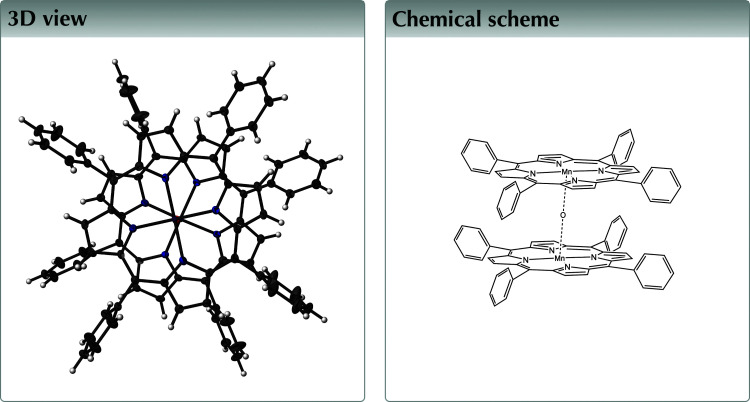

## Structure description

Binuclear manganese species, including bridging oxido ligands, are an essential component in several metalloenzymes (Boal *et al.*, 2012 ▸; Teutloff *et al.*, 2005 ▸; Wieghardt, 1989 ▸). The protonation and deprotonation of the oxido bridge are thought to be important in the catalytic cycle of the redox enzymes (Chen & Yin, 2015 ▸; de Boer *et al.*, 2007 ▸). Scheidt and co-workers previously reported that the manganese(III) μ-hydroxido derivatives {[Mn(OEP)]_2_(OH)}ClO_4_ (OEP = octa­ethyl­porphyrinate) and {[Mn(TPP)]_2_(OH)}ClO_4_ (TPP = tetra­phenyl­porphyrinate) can be prepared by controlled hydrolysis of corresponding monomeric precursor (Cheng *et al.*, 1995 ▸, 1996 ▸). The {[Mn(OEP)]_2_(OH)}ClO_4_ and {[Mn(TPP)]_2_(OH)}ClO_4_ complexes exhibit an average Mn—O distance of 2.011 (18) and 2.026 (1) Å, and an Mn—O(H)—Mn bridging angle of 152.73 (11) and 160.4 (8)°, respectively. The two Mn^III^ ions are displaced by 0.48 and 0.52 Å from their respective 24-atom mean plane. It is inter­esting to note that the μ-oxido species [Mn(OEP)]_2_O is very unstable in halocarbon solvents (Cheng *et al.*, 1995 ▸). In the current report, a new manganese(III) μ-oxido porphyrin derivative, [Mn(TPP)]_2_O, is characterized.

**Figure 1:**
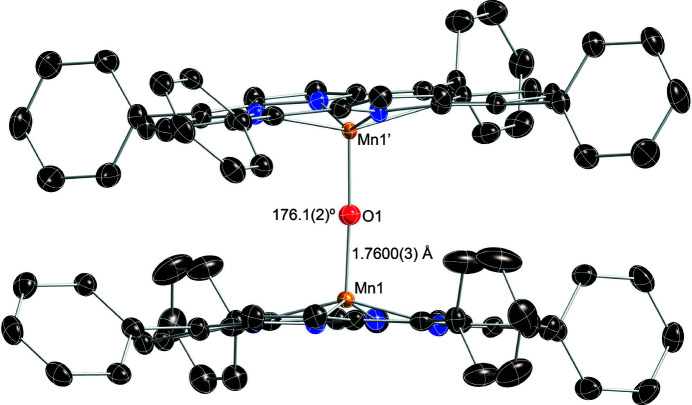
Edge-view of the dinuclear complex of the title compound with displacement elliposids drawn at the 50% probability level. Hydrogen atoms are omitted for clarity.

**Figure 3:**
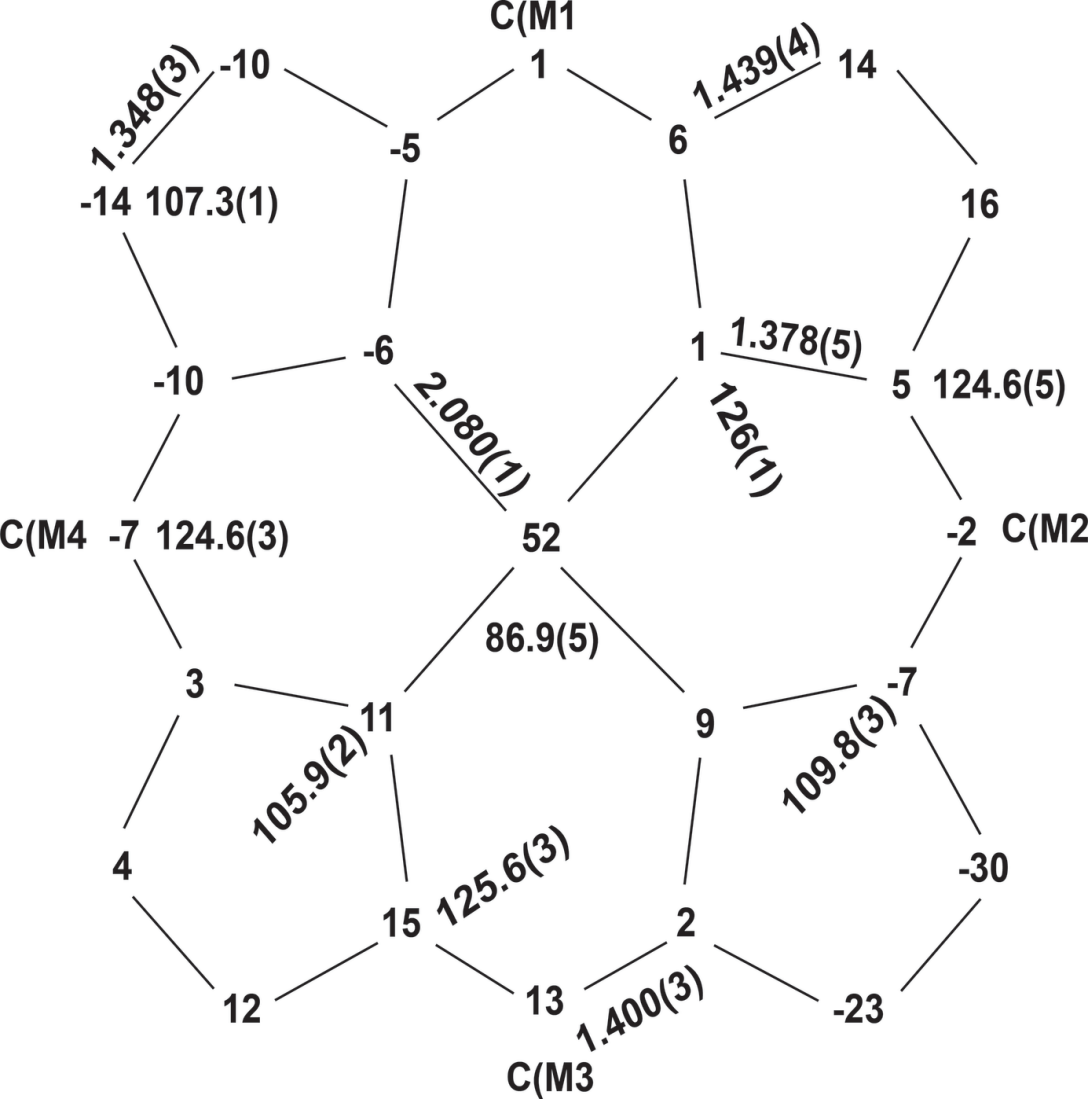
A formal diagram of the porphyrin core of the title compound. Averaged values of the chemically unique bond lengths (Å) and angles (°) are shown. The numbers in parentheses are the e.s.d.s calculated on the assumption that the averaged values were all drawn from the same population. The perpendicular displacements (in units of 0.01 Å) of the porphyrin core atoms from the 24-atom mean plane are also displayed. Positive numbers indicate a displacement toward the central metal atom.

In the crystal structure of the title complex, the asymmetric unit contains one deprotoanted porphyrin mol­ecule located in general position and an oxygen atom on a twofold rotation axis (Wyckoff position 4*a*). Figs. 1 ▸ and 2 ▸ graphically represent the mol­ecular structure of the title μ-oxido complex. As can be seen, the two penta­coordinate manganese(III) ions in [Mn(TPP)]_2_O are bridged by a single oxido ligand with an Mn—O distance of 1.7600 (3) Å and an Mn—O—Mn bridging angle of 176.1 (2)°. The Mn1⋯Mn1′ separation [symmetry code: (’) −*x* + 1, −*y* + 1, *z*) is 3.5180 (5) Å. More qu­anti­tative numerical information is given in Fig. 3 ▸, which contains the detailed displacement of each porphyrin core atom (in units of 0.01 Å) from the 24-atom mean plane. The average Mn^III^—N_porphyrin_ bond length in the porphinato core is 2.080 (1) Å. The manganese atom is displaced by 0.52 Å from its 24-atom mean plane toward the bridging oxido ligand. The average value for the O—Mn—N_porphyrin_ angle is 103 (2)°. The two porphyrin rings are found to be nearly parallel to each other with dihedral angles of 4.08 (8) and 3.68 (8)° between the mean planes of the 24-atom core and the core formed by the four coordinating nitro­gen atoms. In comparison with the reported structure of {[Mn^II^(TPP)]_2_(OH)}ClO_4_, the title compound shows virtually the same metal displacement from the 24-atom mean plane (0.52 Å), while a larger Mn—O—Mn bridging angle [176.1 (2) *versus*. 160.4 (8)°] and a shorter Mn—O distance [1.7600 (3) *versus*. 2.026 (1) Å] is observed.

**Figure 4:**
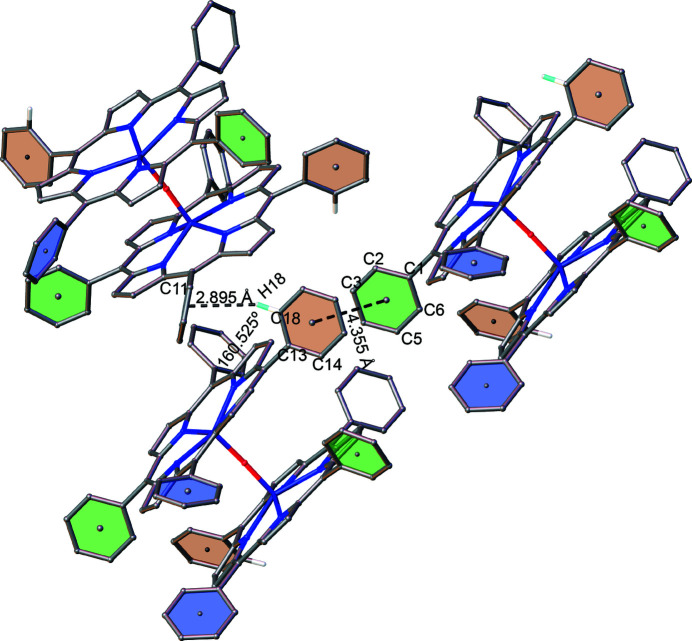
Relevant inter­molecular C—H⋯π and π–π inter­actions in the crystal structure of the title compound.

**Figure 5:**
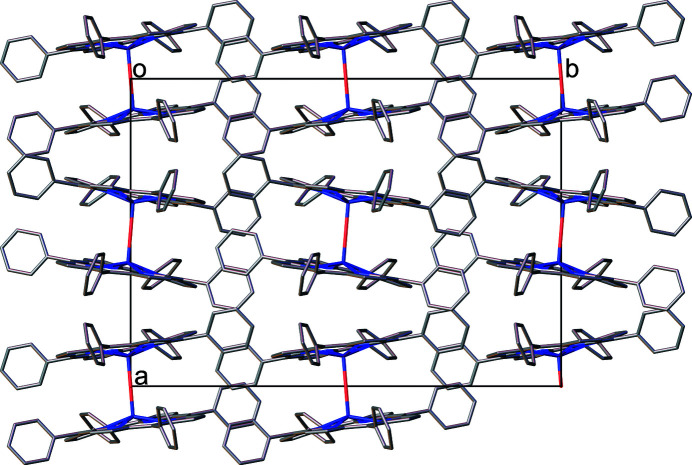
A view of the mol­ecular packing in the crystal structure of the title compound, as seen in a projection along [001]. H atoms are omitted for clarity.

C—H⋯π and π–π inter­actions are found between the packed mol­ecules, which is illustrated in Fig. 4 ▸. As can be seen, the inter­planar distance between the relevant centroids of the rings in the π–π stacking inter­actions is 4.3548 (19) Å, with a slippage of 2.139 Å. The distance between H18 and the relevant centroids of the rings in the C—H⋯π inter­actions is 2.89 Å with an angle of 161°. The mol­ecular packing of the title compound is shown in Fig. 5 ▸.

## Synthesis and crystallization

Unless otherwise noted, all experimental manipulations were performed under argon atmosphere using double-manifold vacuum lines, Schlenk ware and cannula techniques. Except for the solvent used in column chromatography, all solvents used in the experimental process were treated under anhydrous and anaerobic conditions with the pump–freeze–thaw method three times before use. Chloro­benzene and *n*-hexane were distilled over P_2_O_5_ and potassium-sodium alloy, respectively. H_2_TPP and [Mn(TPP)]Cl were prepared according to literature protocols (Adler *et al.*, 1967 ▸; Fleischer *et al.*, 1971 ▸).

The title compound was prepared following a reported procedure (He *et al.*, 2016 ▸). Solid [Mn(TPP)]Cl was dissolved in di­chloro­methane and then shaken vigorously three times with 3 *M* KOH solution. To remove the alkali, the above system was washed with water for an additional two times. To grow single crystals, [Mn(TPP)]_2_O (10 mg) was dissolved in 4 ml of chloro­benzene and cannula-transferred into 8 mm glass tubes, then carefully layered with hexa­nes before sealing the tubes. X-ray quality crystals were obtained several weeks later.

## Refinement

Crystal data, data collection and structure refinement details are summarized in Table 1 ▸. The crystal studied was refined as an inversion twin.

## Supplementary Material

Crystal structure: contains datablock(s) I. DOI: 10.1107/S2414314622008690/wm4172sup1.cif

Structure factors: contains datablock(s) I. DOI: 10.1107/S2414314622008690/wm4172Isup2.hkl

CCDC reference: 2204345

Additional supporting information:  crystallographic information; 3D view; checkCIF report

## Figures and Tables

**Figure 2:**
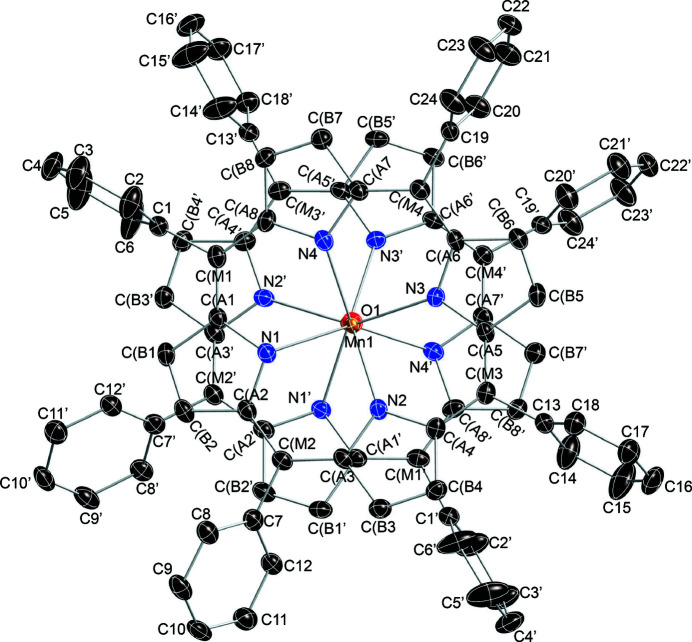
Top-view of the of the dinuclear complex of the title compound with displacement elliposids drawn at the 50% probability level. Hydrogen atoms are omitted for clarity. Primed atoms are generated by symmetry operation −*x* + 1, −*y* + 1, *z*.

**Table 1:** Experimental details

Crystal data	
Chemical formula	[Mn_2_(C_44_H_28_N_4_)_2_O]
*M*_r_	1351.28
Crystal system, space group	Orthorhombic, *A* *e* *a*2
Temperature (K)	100
*a*, *b*, *c* (Å)	17.7931 (6), 24.9494 (10), 15.0943 (6)
*V* (Å^3^)	6700.8 (4)
*Z*	4
Radiation type	Mo *K*α
μ (mm^−1^)	0.43
Crystal size (mm)	0.41 × 0.23 × 0.17

Data collection	
Diffractometer	Bruker APEXII CCD
Absorption correction	Multi-scan (*SADABS*; Krause *et al.*, 2015 ▸)
*T*_min_, *T*_max_	0.678, 0.745
No. of measured, independent and observed [*I* > 2σ(*I*)] reflections	21252, 7046, 6344
*R*_int_	0.032
(sin θ/λ)_max_ (Å^−1^)	0.633

Refinement	
*R*[*F* ^2^ > 2σ(*F* ^2^)], *wR*(*F* ^2^), *S*	0.029, 0.062, 1.05
No. of reflections	7046
No. of parameters	449
No. of restraints	1
H-atom treatment	H-atom parameters constrained
Δρ_max_, Δρ_min_ (e Å^−3^)	0.23, −0.25
Absolute structure	Refined as an inversion twin
Absolute structure parameter	−0.059 (15)

Computer programs: *APEX2* and *SAINT* (Bruker, 2013 ▸), *SHELXS97* (Sheldrick, 2008 ▸), *SHELXL* (Sheldrick, 2015 ▸), *OLEX2* Dolomanov *et al.*, 2009 ▸) and *publCIF* (Westrip, 2010 ▸).

## full crystallographic data

### Crystal data

**Table:**

[Mn_2_(C_44_H_28_N_4_)_2_O]	*D*_x_ = 1.339 Mg m^−^^3^
*M**_r_* = 1351.28	Mo *K*α radiation, λ = 0.71073 Å
Orthorhombic, *A**e**a*2	Cell parameters from 9989 reflections
*a* = 17.7931 (6) Å	θ = 2.7–26.7°
*b* = 24.9494 (10) Å	µ = 0.43 mm^−^^1^
*c* = 15.0943 (6) Å	*T* = 100 K
*V* = 6700.8 (4) Å^3^	Block, black
*Z* = 4	0.41 × 0.23 × 0.17 mm
*F*(000) = 2792	

### Data collection

**Table:**

Bruker APEXII CCD diffractometer	6344 reflections with *I* > 2σ(*I*)
φ and ω scans	*R*_int_ = 0.032
Absorption correction: multi-scan (*SADABS*; Krause *et al.*, 2015)	θ_max_ = 26.8°, θ_min_ = 2.0°
*T*_min_ = 0.678, *T*_max_ = 0.745	*h* = −19→22
21252 measured reflections	*k* = −31→27
7046 independent reflections	*l* = −18→19

### Refinement

**Table:**

Refinement on *F*^2^	H-atom parameters constrained
Least-squares matrix: full	*w* = 1/[σ^2^(*F*_o_^2^) + (0.0273*P*)^2^ + 0.6595*P*] where *P* = (*F*_o_^2^ + 2*F*_c_^2^)/3
*R*[*F*^2^ > 2σ(*F*^2^)] = 0.029	(Δ/σ)_max_ = 0.001
*wR*(*F*^2^) = 0.062	Δρ_max_ = 0.23 e Å^−^^3^
*S* = 1.05	Δρ_min_ = −0.25 e Å^−^^3^
7046 reflections	Extinction correction: SHELXL, Fc^*^=kFc[1+0.001xFc^2^λ^3^/sin(2θ)]^-1/4^
449 parameters	Extinction coefficient: 0.00274 (19)
1 restraint	Absolute structure: Refined as an inversion twin
Hydrogen site location: inferred from neighbouring sites	Absolute structure parameter: −0.059 (15)

### Special details

**Table:**

Geometry. All esds (except the esd in the dihedral angle between two l.s. planes) are estimated using the full covariance matrix. The cell esds are taken into account individually in the estimation of esds in distances, angles and torsion angles; correlations between esds in cell parameters are only used when they are defined by crystal symmetry. An approximate (isotropic) treatment of cell esds is used for estimating esds involving l.s. planes.
Refinement. Refined as a 2-component inversion twin.

### Fractional atomic coordinates and isotropic or equivalent isotropic displacement parameters (Å^2^)

**Table:**

	*x*	*y*	*z*	*U*_iso_*/*U*_eq_	
Mn1	0.59854 (2)	0.49435 (2)	0.50101 (3)	0.01028 (9)	
O1	0.5000	0.5000	0.5050 (2)	0.0183 (5)	
N1	0.61443 (11)	0.45594 (8)	0.38016 (14)	0.0163 (5)	
N2	0.63778 (12)	0.56330 (8)	0.43879 (14)	0.0166 (4)	
N3	0.63396 (11)	0.52824 (8)	0.61960 (14)	0.0160 (4)	
N4	0.61553 (12)	0.42032 (8)	0.56135 (14)	0.0169 (5)	
C1	0.57883 (14)	0.30639 (10)	0.40144 (17)	0.0200 (6)	
C2	0.63612 (16)	0.26904 (11)	0.3961 (2)	0.0341 (7)	
H2	0.6862	0.2796	0.4095	0.041*	
C3	0.62179 (19)	0.21669 (13)	0.3716 (2)	0.0403 (8)	
H3	0.6620	0.1917	0.3679	0.048*	
C4	0.55011 (18)	0.20063 (12)	0.3525 (2)	0.0349 (7)	
H4	0.5404	0.1647	0.3351	0.042*	
C5	0.49248 (19)	0.23687 (14)	0.3588 (3)	0.0510 (10)	
H5	0.4423	0.2257	0.3476	0.061*	
C6	0.50693 (16)	0.28986 (13)	0.3815 (3)	0.0426 (9)	
H6	0.4668	0.3149	0.3833	0.051*	
C7	0.63425 (14)	0.55338 (10)	0.18751 (18)	0.0196 (6)	
C8	0.68001 (14)	0.52864 (11)	0.12510 (16)	0.0213 (6)	
H8	0.7108	0.4993	0.1422	0.026*	
C9	0.68110 (15)	0.54645 (12)	0.03763 (18)	0.0259 (6)	
H9	0.7119	0.5288	−0.0047	0.031*	
C10	0.63767 (15)	0.58949 (11)	0.0123 (2)	0.0271 (6)	
H10	0.6387	0.6016	−0.0473	0.033*	
C11	0.59247 (16)	0.61507 (11)	0.07395 (18)	0.0264 (6)	
H11	0.5629	0.6450	0.0568	0.032*	
C12	0.59039 (14)	0.59682 (10)	0.16089 (17)	0.0211 (6)	
H12	0.5587	0.6141	0.2027	0.025*	
C13	0.66755 (14)	0.67787 (10)	0.59981 (17)	0.0192 (6)	
C14	0.61128 (17)	0.71615 (12)	0.5948 (2)	0.0371 (8)	
H14	0.5629	0.7065	0.5735	0.045*	
C15	0.6253 (2)	0.76868 (12)	0.6209 (3)	0.0487 (10)	
H15	0.5865	0.7947	0.6166	0.058*	
C16	0.69473 (18)	0.78319 (11)	0.6529 (2)	0.0357 (7)	
H16	0.7039	0.8190	0.6714	0.043*	
C17	0.75077 (17)	0.74531 (10)	0.6578 (2)	0.0296 (6)	
H17	0.7990	0.7550	0.6796	0.036*	
C18	0.73730 (15)	0.69298 (10)	0.63100 (18)	0.0252 (6)	
H18	0.7766	0.6672	0.6342	0.030*	
C19	0.63196 (15)	0.43060 (10)	0.81157 (17)	0.0174 (5)	
C20	0.57065 (16)	0.43678 (11)	0.86761 (18)	0.0259 (6)	
H20	0.5252	0.4514	0.8451	0.031*	
C21	0.57487 (17)	0.42202 (12)	0.95577 (19)	0.0305 (7)	
H21	0.5328	0.4271	0.9936	0.037*	
C22	0.64033 (16)	0.39993 (11)	0.9886 (2)	0.0279 (6)	
H22	0.6432	0.3891	1.0489	0.034*	
C23	0.70126 (16)	0.39374 (12)	0.93393 (19)	0.0326 (7)	
H23	0.7465	0.3790	0.9567	0.039*	
C24	0.69735 (15)	0.40887 (12)	0.84570 (18)	0.0285 (6)	
H24	0.7399	0.4043	0.8084	0.034*	
C(A1	0.59878 (14)	0.40270 (11)	0.36534 (17)	0.0169 (6)	
C(A2	0.61536 (14)	0.48008 (11)	0.29787 (17)	0.0175 (5)	
C(A3	0.64614 (14)	0.57202 (10)	0.34861 (17)	0.0172 (6)	
C(A4	0.65444 (13)	0.61138 (10)	0.47894 (16)	0.0178 (6)	
C(A5	0.64461 (14)	0.58203 (11)	0.63499 (16)	0.0175 (6)	
C(A6	0.63481 (14)	0.50402 (10)	0.70127 (17)	0.0163 (6)	
C(A7	0.61839 (14)	0.41017 (11)	0.65087 (17)	0.0173 (6)	
C(A8	0.60215 (13)	0.37132 (10)	0.52147 (17)	0.0177 (6)	
C(B1	0.58917 (14)	0.39337 (10)	0.27237 (17)	0.0206 (6)	
H(B1	0.5787	0.3599	0.2450	0.025*	
C(B2	0.59773 (14)	0.44095 (11)	0.23092 (18)	0.0216 (6)	
H(B2	0.5930	0.4474	0.1691	0.026*	
C(B3	0.67141 (14)	0.62609 (10)	0.33387 (17)	0.0205 (6)	
H(B3	0.6830	0.6418	0.2782	0.025*	
C(B4	0.67566 (13)	0.65027 (10)	0.41336 (17)	0.0193 (5)	
H(B4	0.6900	0.6864	0.4241	0.023*	
C(B5	0.65163 (15)	0.59149 (11)	0.72899 (17)	0.0214 (6)	
H(B5	0.6588	0.6252	0.7571	0.026*	
C(B6	0.64606 (15)	0.54341 (10)	0.76930 (18)	0.0212 (6)	
H(B6	0.6490	0.5368	0.8312	0.025*	
C(B7	0.60762 (14)	0.35390 (10)	0.66730 (18)	0.0219 (6)	
H(B7	0.6077	0.3368	0.7235	0.026*	
C(B8	0.59734 (15)	0.33004 (11)	0.58816 (18)	0.0212 (6)	
H(B8	0.5886	0.2930	0.5781	0.025*	
C(M1	0.59397 (13)	0.36267 (10)	0.43050 (17)	0.0181 (5)	
C(M2	0.63186 (14)	0.53413 (10)	0.28190 (18)	0.0171 (5)	
C(M3	0.65360 (14)	0.62145 (10)	0.56998 (17)	0.0176 (5)	
C(M4	0.62837 (14)	0.44908 (10)	0.71700 (17)	0.0171 (6)	

### Atomic displacement parameters (Å^2^)

**Table:**

	*U*^11^	*U*^22^	*U*^33^	*U*^12^	*U*^13^	*U*^23^
Mn1	0.01042 (14)	0.01132 (17)	0.00910 (15)	0.00002 (12)	−0.00037 (17)	−0.00131 (17)
O1	0.0170 (10)	0.0199 (11)	0.0180 (11)	0.0008 (9)	0.000	0.000
N1	0.0180 (10)	0.0171 (11)	0.0137 (12)	0.0010 (8)	0.0005 (9)	−0.0010 (9)
N2	0.0175 (11)	0.0181 (11)	0.0142 (11)	−0.0016 (9)	−0.0006 (9)	−0.0026 (9)
N3	0.0174 (10)	0.0150 (11)	0.0156 (12)	−0.0016 (9)	−0.0009 (9)	−0.0011 (9)
N4	0.0172 (11)	0.0193 (11)	0.0142 (11)	0.0015 (8)	−0.0007 (8)	−0.0015 (9)
C1	0.0250 (13)	0.0190 (14)	0.0160 (13)	−0.0013 (11)	−0.0002 (11)	−0.0011 (11)
C2	0.0252 (14)	0.0251 (15)	0.052 (2)	0.0011 (12)	−0.0058 (15)	−0.0135 (14)
C3	0.0412 (19)	0.0241 (16)	0.056 (2)	0.0065 (14)	−0.0102 (16)	−0.0148 (15)
C4	0.0436 (18)	0.0206 (15)	0.0406 (18)	−0.0059 (13)	0.0022 (14)	−0.0093 (13)
C5	0.0302 (18)	0.043 (2)	0.080 (3)	−0.0106 (15)	0.0043 (17)	−0.0316 (19)
C6	0.0236 (15)	0.0313 (17)	0.073 (3)	−0.0005 (12)	0.0015 (16)	−0.0262 (17)
C7	0.0182 (13)	0.0219 (14)	0.0187 (15)	−0.0048 (11)	−0.0009 (11)	−0.0011 (11)
C8	0.0174 (12)	0.0272 (14)	0.0193 (14)	−0.0031 (11)	−0.0009 (11)	−0.0014 (11)
C9	0.0250 (14)	0.0357 (16)	0.0168 (13)	−0.0059 (12)	0.0049 (11)	−0.0036 (11)
C10	0.0327 (15)	0.0347 (15)	0.0140 (15)	−0.0077 (11)	−0.0023 (13)	0.0040 (13)
C11	0.0289 (15)	0.0268 (15)	0.0236 (15)	−0.0019 (12)	−0.0068 (12)	0.0020 (12)
C12	0.0207 (13)	0.0246 (14)	0.0180 (14)	−0.0011 (10)	−0.0003 (11)	−0.0002 (11)
C13	0.0235 (13)	0.0192 (14)	0.0150 (12)	−0.0020 (11)	−0.0006 (10)	−0.0012 (10)
C14	0.0287 (16)	0.0281 (17)	0.054 (2)	0.0050 (13)	−0.0121 (15)	−0.0153 (15)
C15	0.047 (2)	0.0242 (17)	0.075 (3)	0.0119 (15)	−0.0185 (19)	−0.0161 (18)
C16	0.0488 (19)	0.0177 (14)	0.0405 (18)	−0.0043 (13)	−0.0099 (15)	−0.0064 (13)
C17	0.0316 (15)	0.0279 (16)	0.0294 (15)	−0.0100 (13)	−0.0027 (12)	−0.0039 (12)
C18	0.0265 (15)	0.0236 (14)	0.0255 (14)	−0.0024 (11)	−0.0008 (12)	−0.0022 (11)
C19	0.0198 (13)	0.0182 (13)	0.0142 (13)	−0.0024 (10)	0.0005 (10)	−0.0017 (10)
C20	0.0201 (13)	0.0352 (17)	0.0224 (15)	0.0035 (12)	0.0018 (11)	0.0039 (12)
C21	0.0295 (16)	0.0397 (17)	0.0224 (15)	0.0003 (14)	0.0100 (12)	0.0039 (13)
C22	0.0382 (16)	0.0288 (15)	0.0168 (16)	0.0017 (11)	0.0005 (13)	0.0035 (12)
C23	0.0291 (16)	0.0488 (19)	0.0199 (14)	0.0099 (14)	−0.0055 (12)	0.0044 (13)
C24	0.0232 (14)	0.0415 (17)	0.0207 (14)	0.0047 (13)	0.0017 (11)	0.0021 (12)
C(A1	0.0164 (13)	0.0188 (14)	0.0156 (14)	0.0014 (10)	0.0002 (10)	−0.0030 (11)
C(A2	0.0174 (13)	0.0204 (14)	0.0146 (14)	0.0022 (11)	0.0010 (10)	−0.0024 (11)
C(A3	0.0152 (12)	0.0221 (15)	0.0143 (13)	0.0001 (10)	0.0015 (10)	0.0002 (11)
C(A4	0.0147 (12)	0.0181 (13)	0.0205 (15)	−0.0006 (10)	−0.0002 (10)	−0.0023 (10)
C(A5	0.0187 (13)	0.0202 (14)	0.0137 (13)	−0.0002 (11)	−0.0010 (10)	−0.0033 (11)
C(A6	0.0168 (13)	0.0197 (15)	0.0125 (13)	−0.0003 (10)	0.0004 (10)	−0.0030 (10)
C(A7	0.0186 (13)	0.0182 (14)	0.0151 (14)	0.0039 (11)	−0.0014 (10)	0.0001 (10)
C(A8	0.0174 (12)	0.0139 (12)	0.0217 (16)	0.0021 (9)	−0.0014 (10)	−0.0036 (10)
C(B1	0.0233 (14)	0.0209 (14)	0.0177 (13)	−0.0017 (11)	−0.0015 (10)	−0.0053 (11)
C(B2	0.0226 (14)	0.0270 (15)	0.0151 (14)	−0.0004 (11)	−0.0005 (11)	−0.0030 (11)
C(B3	0.0202 (13)	0.0215 (14)	0.0198 (14)	−0.0008 (11)	0.0014 (11)	0.0035 (11)
C(B4	0.0193 (12)	0.0176 (13)	0.0209 (14)	−0.0031 (10)	−0.0003 (10)	0.0009 (11)
C(B5	0.0254 (14)	0.0205 (14)	0.0184 (14)	−0.0016 (11)	0.0002 (11)	−0.0066 (11)
C(B6	0.0274 (14)	0.0220 (15)	0.0141 (14)	−0.0008 (12)	0.0000 (11)	−0.0019 (11)
C(B7	0.0261 (14)	0.0193 (14)	0.0205 (14)	0.0021 (11)	−0.0017 (11)	0.0003 (11)
C(B8	0.0267 (14)	0.0151 (14)	0.0217 (14)	0.0004 (11)	−0.0020 (11)	0.0005 (11)
C(M1	0.0167 (13)	0.0197 (14)	0.0179 (14)	0.0021 (10)	−0.0007 (10)	−0.0040 (11)
C(M2	0.0150 (12)	0.0210 (14)	0.0152 (14)	0.0004 (11)	0.0002 (10)	0.0011 (11)
C(M3	0.0138 (12)	0.0181 (13)	0.0211 (14)	0.0002 (10)	−0.0005 (11)	−0.0033 (11)
C(M4	0.0152 (12)	0.0198 (14)	0.0162 (14)	0.0006 (10)	−0.0007 (10)	0.0002 (11)

### Geometric parameters (Å, º)

**Table:**

Mn1—O1	1.7600 (3)	C16—H16	0.9500
Mn1—N1	2.080 (2)	C16—C17	1.376 (4)
Mn1—N2	2.081 (2)	C17—H17	0.9500
Mn1—N3	2.078 (2)	C17—C18	1.388 (3)
Mn1—N4	2.081 (2)	C18—H18	0.9500
O1—Mn1^i^	1.7599 (3)	C19—C20	1.389 (4)
N1—C(A1	1.375 (4)	C19—C24	1.383 (4)
N1—C(A2	1.380 (3)	C19—C(M4	1.502 (4)
N2—C(A3	1.386 (3)	C20—H20	0.9500
N2—C(A4	1.376 (3)	C20—C21	1.383 (4)
N3—C(A5	1.375 (3)	C21—H21	0.9500
N3—C(A6	1.373 (3)	C21—C22	1.381 (4)
N4—C(A7	1.376 (3)	C22—H22	0.9500
N4—C(A8	1.383 (3)	C22—C23	1.371 (4)
C1—C2	1.384 (4)	C23—H23	0.9500
C1—C6	1.377 (4)	C23—C24	1.386 (4)
C1—C(M1	1.495 (4)	C24—H24	0.9500
C2—H2	0.9500	C(A1—C(B1	1.433 (4)
C2—C3	1.381 (4)	C(A1—C(M1	1.404 (4)
C3—H3	0.9500	C(A2—C(B2	1.440 (4)
C3—C4	1.367 (4)	C(A2—C(M2	1.401 (4)
C4—H4	0.9500	C(A3—C(B3	1.439 (4)
C4—C5	1.370 (5)	C(A3—C(M2	1.404 (4)
C5—H5	0.9500	C(A4—C(B4	1.436 (3)
C5—C6	1.390 (4)	C(A4—C(M3	1.397 (4)
C6—H6	0.9500	C(A5—C(B5	1.444 (4)
C7—C8	1.390 (4)	C(A5—C(M3	1.399 (4)
C7—C12	1.395 (4)	C(A6—C(B6	1.435 (4)
C7—C(M2	1.504 (4)	C(A6—C(M4	1.396 (3)
C8—H8	0.9500	C(A7—C(B7	1.438 (4)
C8—C9	1.393 (4)	C(A7—C(M4	1.404 (4)
C9—H9	0.9500	C(A8—C(B8	1.443 (4)
C9—C10	1.377 (4)	C(A8—C(M1	1.398 (4)
C10—H10	0.9500	C(B1—H(B1	0.9500
C10—C11	1.385 (4)	C(B1—C(B2	1.351 (4)
C11—H11	0.9500	C(B2—H(B2	0.9500
C11—C12	1.390 (4)	C(B3—H(B3	0.9500
C12—H12	0.9500	C(B3—C(B4	1.345 (4)
C13—C14	1.386 (4)	C(B4—H(B4	0.9500
C13—C18	1.380 (3)	C(B5—H(B5	0.9500
C13—C(M3	1.499 (3)	C(B5—C(B6	1.349 (4)
C14—H14	0.9500	C(B6—H(B6	0.9500
C14—C15	1.391 (4)	C(B7—H(B7	0.9500
C15—H15	0.9500	C(B7—C(B8	1.347 (4)
C15—C16	1.375 (4)	C(B8—H(B8	0.9500
			
O1—Mn1—N1	101.66 (12)	C24—C19—C20	118.5 (2)
O1—Mn1—N2	106.46 (8)	C24—C19—C(M4	120.7 (2)
O1—Mn1—N3	103.90 (12)	C19—C20—H20	119.5
O1—Mn1—N4	101.59 (8)	C21—C20—C19	120.9 (3)
N1—Mn1—N2	86.53 (8)	C21—C20—H20	119.5
N1—Mn1—N4	87.44 (8)	C20—C21—H21	120.1
N2—Mn1—N4	151.94 (9)	C22—C21—C20	119.9 (3)
N3—Mn1—N1	154.41 (8)	C22—C21—H21	120.1
N3—Mn1—N2	87.17 (8)	C21—C22—H22	120.1
N3—Mn1—N4	86.56 (8)	C23—C22—C21	119.7 (3)
Mn1^i^—O1—Mn1	176.1 (2)	C23—C22—H22	120.1
C(A1—N1—Mn1	124.06 (16)	C22—C23—H23	119.7
C(A1—N1—C(A2	106.1 (2)	C22—C23—C24	120.5 (3)
C(A2—N1—Mn1	126.14 (17)	C24—C23—H23	119.7
C(A3—N2—Mn1	127.51 (17)	C19—C24—C23	120.5 (3)
C(A4—N2—Mn1	126.46 (17)	C19—C24—H24	119.8
C(A4—N2—C(A3	105.8 (2)	C23—C24—H24	119.8
C(A5—N3—Mn1	125.77 (17)	N1—C(A1—C(B1	109.9 (2)
C(A6—N3—Mn1	126.71 (16)	N1—C(A1—C(M1	125.8 (2)
C(A6—N3—C(A5	106.0 (2)	C(M1—C(A1—C(B1	124.3 (2)
C(A7—N4—Mn1	126.77 (17)	N1—C(A2—C(B2	109.5 (2)
C(A7—N4—C(A8	105.7 (2)	N1—C(A2—C(M2	125.3 (2)
C(A8—N4—Mn1	124.67 (16)	C(M2—C(A2—C(B2	125.3 (2)
C2—C1—C(M1	121.1 (2)	N2—C(A3—C(B3	109.4 (2)
C6—C1—C2	118.0 (3)	N2—C(A3—C(M2	125.4 (2)
C6—C1—C(M1	120.8 (2)	C(M2—C(A3—C(B3	125.2 (2)
C1—C2—H2	119.4	N2—C(A4—C(B4	110.0 (2)
C3—C2—C1	121.1 (3)	N2—C(A4—C(M3	126.0 (2)
C3—C2—H2	119.4	C(M3—C(A4—C(B4	124.0 (2)
C2—C3—H3	119.8	N3—C(A5—C(B5	109.7 (2)
C4—C3—C2	120.4 (3)	N3—C(A5—C(M3	125.7 (2)
C4—C3—H3	119.8	C(M3—C(A5—C(B5	124.4 (2)
C3—C4—H4	120.3	N3—C(A6—C(B6	110.0 (2)
C3—C4—C5	119.3 (3)	N3—C(A6—C(M4	125.7 (2)
C5—C4—H4	120.3	C(M4—C(A6—C(B6	124.2 (2)
C4—C5—H5	119.8	N4—C(A7—C(B7	110.1 (2)
C4—C5—C6	120.4 (3)	N4—C(A7—C(M4	125.2 (2)
C6—C5—H5	119.8	C(M4—C(A7—C(B7	124.7 (2)
C1—C6—C5	120.7 (3)	N4—C(A8—C(B8	109.7 (2)
C1—C6—H6	119.6	N4—C(A8—C(M1	125.6 (2)
C5—C6—H6	119.6	C(M1—C(A8—C(B8	124.7 (2)
C8—C7—C12	118.5 (2)	C(A1—C(B1—H(B1	126.4
C8—C7—C(M2	121.1 (2)	C(B2—C(B1—C(A1	107.3 (2)
C12—C7—C(M2	120.4 (2)	C(B2—C(B1—H(B1	126.4
C7—C8—H8	119.7	C(A2—C(B2—H(B2	126.4
C7—C8—C9	120.6 (3)	C(B1—C(B2—C(A2	107.2 (2)
C9—C8—H8	119.7	C(B1—C(B2—H(B2	126.4
C8—C9—H9	119.9	C(A3—C(B3—H(B3	126.3
C10—C9—C8	120.2 (3)	C(B4—C(B3—C(A3	107.5 (2)
C10—C9—H9	119.9	C(B4—C(B3—H(B3	126.3
C9—C10—H10	120.0	C(A4—C(B4—H(B4	126.4
C9—C10—C11	119.9 (3)	C(B3—C(B4—C(A4	107.3 (2)
C11—C10—H10	120.0	C(B3—C(B4—H(B4	126.4
C10—C11—H11	120.1	C(A5—C(B5—H(B5	126.5
C10—C11—C12	119.9 (3)	C(B6—C(B5—C(A5	107.0 (2)
C12—C11—H11	120.1	C(B6—C(B5—H(B5	126.5
C7—C12—H12	119.6	C(A6—C(B6—H(B6	126.4
C11—C12—C7	120.8 (2)	C(B5—C(B6—C(A6	107.2 (2)
C11—C12—H12	119.6	C(B5—C(B6—H(B6	126.4
C14—C13—C(M3	120.8 (2)	C(A7—C(B7—H(B7	126.4
C18—C13—C14	118.7 (3)	C(B8—C(B7—C(A7	107.3 (2)
C18—C13—C(M3	120.5 (2)	C(B8—C(B7—H(B7	126.4
C13—C14—H14	119.8	C(A8—C(B8—H(B8	126.4
C13—C14—C15	120.3 (3)	C(B7—C(B8—C(A8	107.2 (2)
C15—C14—H14	119.8	C(B7—C(B8—H(B8	126.4
C14—C15—H15	119.7	C(A1—C(M1—C1	118.2 (2)
C16—C15—C14	120.6 (3)	C(A8—C(M1—C1	116.9 (2)
C16—C15—H15	119.7	C(A8—C(M1—C(A1	124.9 (2)
C15—C16—H16	120.4	C(A2—C(M2—C7	118.4 (2)
C15—C16—C17	119.3 (3)	C(A2—C(M2—C(A3	124.2 (2)
C17—C16—H16	120.4	C(A3—C(M2—C7	117.3 (2)
C16—C17—H17	119.8	C(A4—C(M3—C13	117.6 (2)
C16—C17—C18	120.4 (3)	C(A4—C(M3—C(A5	124.4 (2)
C18—C17—H17	119.8	C(A5—C(M3—C13	117.9 (2)
C13—C18—C17	120.8 (3)	C(A6—C(M4—C19	117.4 (2)
C13—C18—H18	119.6	C(A6—C(M4—C(A7	124.7 (2)
C17—C18—H18	119.6	C(A7—C(M4—C19	118.0 (2)
C20—C19—C(M4	120.8 (2)		
			
Mn1—N1—C(A1—C(B1	158.96 (17)	C16—C17—C18—C13	−0.6 (4)
Mn1—N1—C(A1—C(M1	−22.0 (3)	C18—C13—C14—C15	0.0 (5)
Mn1—N1—C(A2—C(B2	−157.21 (17)	C18—C13—C(M3—C(A4	−100.6 (3)
Mn1—N1—C(A2—C(M2	23.5 (3)	C18—C13—C(M3—C(A5	76.1 (3)
Mn1—N2—C(A3—C(B3	177.13 (16)	C19—C20—C21—C22	−1.2 (5)
Mn1—N2—C(A3—C(M2	−2.6 (4)	C20—C19—C24—C23	−0.1 (4)
Mn1—N2—C(A4—C(B4	−176.57 (16)	C20—C19—C(M4—C(A6	76.7 (3)
Mn1—N2—C(A4—C(M3	5.3 (4)	C20—C19—C(M4—C(A7	−103.0 (3)
Mn1—N3—C(A5—C(B5	166.36 (17)	C20—C21—C22—C23	1.3 (5)
Mn1—N3—C(A5—C(M3	−18.5 (4)	C21—C22—C23—C24	−0.8 (5)
Mn1—N3—C(A6—C(B6	−166.57 (17)	C22—C23—C24—C19	0.2 (5)
Mn1—N3—C(A6—C(M4	15.5 (4)	C24—C19—C20—C21	0.5 (4)
Mn1—N4—C(A7—C(B7	162.21 (17)	C24—C19—C(M4—C(A6	−100.9 (3)
Mn1—N4—C(A7—C(M4	−16.2 (4)	C24—C19—C(M4—C(A7	79.4 (3)
Mn1—N4—C(A8—C(B8	−162.46 (16)	C(A1—N1—C(A2—C(B2	1.7 (3)
Mn1—N4—C(A8—C(M1	17.2 (3)	C(A1—N1—C(A2—C(M2	−177.6 (2)
N1—C(A1—C(B1—C(B2	−1.0 (3)	C(A1—C(B1—C(B2—C(A2	1.9 (3)
N1—C(A1—C(M1—C1	−178.0 (2)	C(A2—N1—C(A1—C(B1	−0.5 (3)
N1—C(A1—C(M1—C(A8	2.1 (4)	C(A2—N1—C(A1—C(M1	178.5 (2)
N1—C(A2—C(B2—C(B1	−2.3 (3)	C(A3—N2—C(A4—C(B4	−1.7 (3)
N1—C(A2—C(M2—C7	176.9 (2)	C(A3—N2—C(A4—C(M3	−179.8 (2)
N1—C(A2—C(M2—C(A3	−3.4 (4)	C(A3—C(B3—C(B4—C(A4	1.0 (3)
N2—C(A3—C(B3—C(B4	−2.2 (3)	C(A4—N2—C(A3—C(B3	2.3 (3)
N2—C(A3—C(M2—C7	172.0 (2)	C(A4—N2—C(A3—C(M2	−177.4 (2)
N2—C(A3—C(M2—C(A2	−7.7 (4)	C(A5—N3—C(A6—C(B6	0.1 (3)
N2—C(A4—C(B4—C(B3	0.4 (3)	C(A5—N3—C(A6—C(M4	−177.8 (2)
N2—C(A4—C(M3—C13	−175.2 (2)	C(A5—C(B5—C(B6—C(A6	−0.6 (3)
N2—C(A4—C(M3—C(A5	8.3 (4)	C(A6—N3—C(A5—C(B5	−0.5 (3)
N3—C(A5—C(B5—C(B6	0.7 (3)	C(A6—N3—C(A5—C(M3	174.6 (2)
N3—C(A5—C(M3—C13	−177.7 (2)	C(A7—N4—C(A8—C(B8	−0.5 (3)
N3—C(A5—C(M3—C(A4	−1.3 (4)	C(A7—N4—C(A8—C(M1	179.1 (2)
N3—C(A6—C(B6—C(B5	0.3 (3)	C(A7—C(B7—C(B8—C(A8	0.4 (3)
N3—C(A6—C(M4—C19	179.0 (2)	C(A8—N4—C(A7—C(B7	0.7 (3)
N3—C(A6—C(M4—C(A7	−1.4 (4)	C(A8—N4—C(A7—C(M4	−177.7 (2)
N4—C(A7—C(B7—C(B8	−0.7 (3)	C(B1—C(A1—C(M1—C1	0.8 (4)
N4—C(A7—C(M4—C19	−178.6 (2)	C(B1—C(A1—C(M1—C(A8	−179.0 (2)
N4—C(A7—C(M4—C(A6	1.8 (4)	C(B2—C(A2—C(M2—C7	−2.2 (4)
N4—C(A8—C(B8—C(B7	0.1 (3)	C(B2—C(A2—C(M2—C(A3	177.5 (3)
N4—C(A8—C(M1—C1	−179.3 (2)	C(B3—C(A3—C(M2—C7	−7.7 (4)
N4—C(A8—C(M1—C(A1	0.6 (4)	C(B3—C(A3—C(M2—C(A2	172.6 (2)
C1—C2—C3—C4	−0.4 (5)	C(B4—C(A4—C(M3—C13	7.0 (4)
C2—C1—C6—C5	1.5 (5)	C(B4—C(A4—C(M3—C(A5	−169.5 (2)
C2—C1—C(M1—C(A1	101.4 (3)	C(B5—C(A5—C(M3—C13	−3.3 (4)
C2—C1—C(M1—C(A8	−78.8 (3)	C(B5—C(A5—C(M3—C(A4	173.2 (3)
C2—C3—C4—C5	−0.6 (5)	C(B6—C(A6—C(M4—C19	1.3 (4)
C3—C4—C5—C6	2.1 (6)	C(B6—C(A6—C(M4—C(A7	−179.1 (3)
C4—C5—C6—C1	−2.5 (6)	C(B7—C(A7—C(M4—C19	3.2 (4)
C6—C1—C2—C3	−0.1 (5)	C(B7—C(A7—C(M4—C(A6	−176.4 (2)
C6—C1—C(M1—C(A1	−80.4 (4)	C(B8—C(A8—C(M1—C1	0.3 (4)
C6—C1—C(M1—C(A8	99.5 (3)	C(B8—C(A8—C(M1—C(A1	−179.8 (2)
C7—C8—C9—C10	1.1 (4)	C(M1—C1—C2—C3	178.2 (3)
C8—C7—C12—C11	−0.1 (4)	C(M1—C1—C6—C5	−176.8 (3)
C8—C7—C(M2—C(A2	−54.8 (3)	C(M1—C(A1—C(B1—C(B2	−180.0 (2)
C8—C7—C(M2—C(A3	125.5 (3)	C(M1—C(A8—C(B8—C(B7	−179.6 (2)
C8—C9—C10—C11	−0.3 (4)	C(M2—C7—C8—C9	179.3 (2)
C9—C10—C11—C12	−0.8 (4)	C(M2—C7—C12—C11	179.7 (2)
C10—C11—C12—C7	1.0 (4)	C(M2—C(A2—C(B2—C(B1	176.9 (2)
C12—C7—C8—C9	−0.9 (4)	C(M2—C(A3—C(B3—C(B4	177.6 (2)
C12—C7—C(M2—C(A2	125.4 (3)	C(M3—C13—C14—C15	−178.6 (3)
C12—C7—C(M2—C(A3	−54.3 (3)	C(M3—C13—C18—C17	179.3 (3)
C13—C14—C15—C16	−0.7 (6)	C(M3—C(A4—C(B4—C(B3	178.6 (2)
C14—C13—C18—C17	0.7 (4)	C(M3—C(A5—C(B5—C(B6	−174.5 (2)
C14—C13—C(M3—C(A4	78.0 (3)	C(M4—C19—C20—C21	−177.1 (3)
C14—C13—C(M3—C(A5	−105.3 (3)	C(M4—C19—C24—C23	177.5 (3)
C14—C15—C16—C17	0.9 (6)	C(M4—C(A6—C(B6—C(B5	178.3 (2)
C15—C16—C17—C18	−0.2 (5)	C(M4—C(A7—C(B7—C(B8	177.7 (2)

Symmetry code: (i) −*x*+1, −*y*+1, *z*.
